# Frequency and anatomic variability of the mandibular lingual foramina: a cone-beam CT study

**DOI:** 10.1186/s12880-022-00736-2

**Published:** 2022-01-20

**Authors:** Silvio Taschieri, Stefano Corbella, Amel Silnovic, Luca Francetti, Carmelo Messina, Luca Maria Sconfienza, Domenico Albano

**Affiliations:** 1 Department of Biomedical, Surgical and Dental Sciences, Università Degli Studi Di Milano, Milan, Italy; 2 IRCCS Istituto Ortopedico Galeazzi, Milan, Italy; 3 Department of Oral Surgery, Institute of Dentistry, I. M. Sechenov First Moscow State Medical University, Moscow, Russia; 4 Dipartimento Di Scienze Biomediche Per La Salute, Università Degli Studi Di Milano, Milan, Italy; 5 Sezione Di Scienze Radiologiche, Dipartimento Di Biomedicina, Neuroscienze E Diagnostica Avanzata, Università Degli Studi Di Palermo, Palermo, Italy

**Keywords:** Cone-beam computed tomography, Lingual foramen, Mandible, Anatomy, Vascularization

## Abstract

**Background:**

To evaluate the distribution of lingual foramina (LF) and their correlation with demographic characteristics and mandible width, shape, and bone thickness in Caucasian Italian patients subjected to cone-beam CT (CBCT).

**Methods:**

CBCTs were reviewed to assess the number of all LF, midline and lateral LF. We also assessed the relationship of the number of lateral LF with gender and mandibular width, shape, and bone thickness using the Chi Square test. A *p* value < 0.05 was considered statistically significant.

**Results:**

Three-hundred patients (180 males; age range: 21–87 years) were included. The highest frequency per patient was of 2 LF (97/300, 32.3%), followed by 3 (81/300, 27%) and 4 (53/300, 17.7%). No LF were observed in 2/300 patients (0.7%), while the highest number was of 8 LF in one patient. The highest frequency of midline LF per person was of 2 LF (57.3%, 172/300), while the highest number per person was 5 LF in one patient (0.3%). The highest frequency of midline LF located above and below the genial tubercle was of 1 in 197/300 patients (65.7%) and in 169/300 patients (56.3%), respectively. Concerning lateral LF, the highest frequencies were of 0 (113/300, 37.7%) and of 1 (112/300, 37.3%). We did not observe any significant difference of the number of midline and lateral LF based on gender (*P* = .438 and *P* = .195, respectively) or mandible width (*P* = .069 and *P* = .114, respectively). The mandible shape was normal in 188 cases, with facial constriction in 42, lingual constriction in 54, and hour glass constriction in 16. The mean bone thickness was 10.76 mm in the symphysis, 10.92 mm in the right hemiarches, and 10.68 in the left hemiarches. No significant differences in the distribution of LF were observed also based on mandibular shape and bone thickness (both with *P* > .05).

**Conclusions:**

We have shown the high variability of number and anatomic distribution of LF in an Italian group of patients subjected to CBCT without reporting any association with gender and mandible width, shape, and bone thickness.

## Background

The lingual foramina (LF) host important vascular and neural structures coming from the floor of mouth that perforate the cortical bone of the mandible on the lingual side providing vascular and nerve supply to the mental region [1]. Specifically, the sublingual artery, which is a branch of the lingual artery, travels along the superior face of the mylohyoid muscle and then through the LF and anastomoses with central inferior alveolar vessels, while the submental artery, which is a branch of the facial artery, travels along the inferior face of the mylohyoid muscle and then penetrates the mental region to anastomose with branches of the anterior alveolar artery [2, 3]. An extreme variability has been reported in the number and topographic distribution of LF and in the type and number of anastomoses between these two arteries. Despite the complex vascularization of the floor of mouth and mental region, there is a diffuse mistaken belief among oral surgeons that the mandibular region included between the mental foramina is the safest for surgery procedures. Unfortunately, the proof of this misconception is the non-negligible number of massive bleeding accidents having been reported after implant interventions in this region [4–8]. Such cases, even though not so frequent as compared to the total number of implants placed, could be a significant life hazard. For such reason, a detailed anatomic knowledge of the vascularization of the interforaminal area as well as a thorough pre-operative imaging evaluation of the variability of LF are essential to prevent life-threatening complications during surgical procedures. Indeed, the individual anatomical variability of mandibular neuro-vascular bundles is a crucial factor to be considered when dealing with mandibular surgery [9]. Previous cadaveric and imaging studies, mostly based on computed tomography (CT) examinations, have investigated this point highlighting the extreme anatomic variability between different populations [10]. The aim our study was to evaluate the number and position of LF and to evaluate if correlation exists with demographic characteristics and mandible width, shape, and bone thickness in a sample made of Caucasian Italian patients subjected to cone-beam CT (CBCT).

## Methods

This retrospective study was approved by our Institutional Review Board and the requirement for informed consent was waived. After matching imaging and demographic data, our dataset was anonymized to remove any connections between data and patients’ identity according to the General Data Protection Regulation for Research Hospitals.

### Patient population

This study is concerned with the evaluation of anatomic variability of LF in a consecutive series of Caucasian Italian patients subjected to CBCT at our Institution from January 2017 to December 2020. CBCT scans were done for several reasons, including pre-operative planning for implant or dental surgery. We included in our series all Caucasian Italian patients with permanent (non-mixed or deciduous) teeth with age ≥ 20 years. The following exclusion criteria were applied: (i) edentulism, specifically the absence of mandibular central/lateral incisors and canines, since mandible morphology may vary substantially in patients with this condition; (ii) motion artifacts such that compromise images quality making not possible the evaluation of LF; (iii) previous mandibular surgery like osteotomy; (iv) bony lesions of the mandible; (v) mandibular fractures.

### CBCT and images interpretation

CBCT scans were done on a 3D Accuitomo XYZ Slice View Tomograph® (Model MCT-1, Type EX-1/EX-2; Fushimi-ku, Kyoto: J. Morita Mfg. Corp). The acquisition protocol included the following imaging parameters: tube voltage, 60–80 kV; tube current, 1–10 mA; a voxel size of 0.125 × 0.125 × 0.125 mm. First, the measurements were taken by an undergraduate dentistry student with 4 years of experience in CBCT imaging, previously calibrated with a board-certified experienced radiologist (D.A.), with 8 years of experience in CBCT imaging, by examining together 30 sample scans, until reaching a concordance in measurements. Both reviewers were blinded with patients age and gender. Even after calibration, the entire process of images review was supervised by a radiologist (D.A.), but only one final set of measurements were obtained by the undergraduate dentistry student. Notably, the high reproducibility of these measurements has been already proven by previous studies [11, 12]. We have reviewed all images in a liquid crystal display monitor backlit with cold-cathode fluorescent lamps.

**Fig. 1:**
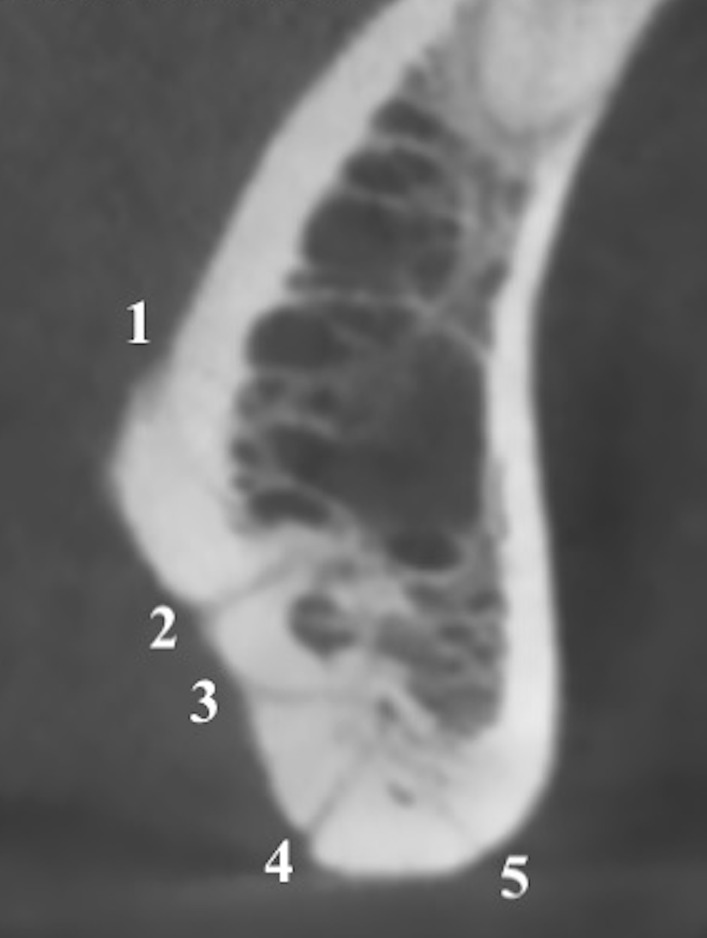
Sagittal multiplanar reconstruction of the symphysis showing 5 midline LF
- the number and position of midline LF in the middle point of the symphysis differentiating those located above the genial tubercle (generally considered as the foramen of the sublingual artery) from those located below it (generally considered as the foramen of the submental artery) (Fig. 2);

All scans were evaluated to assess:

- the number of all LF, including both midline and lateral foramina (Fig. 1);

**Fig. 2:**
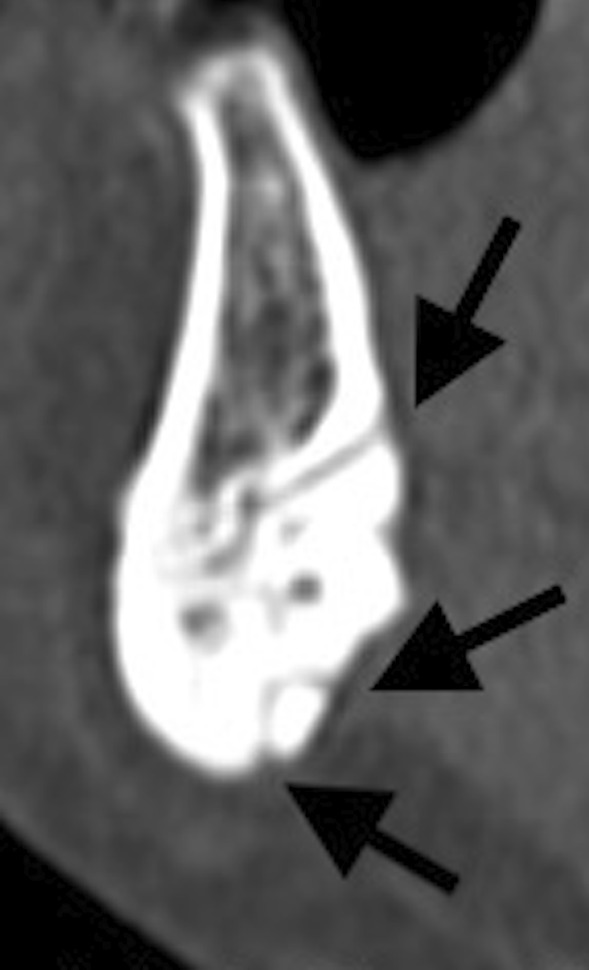
Sagittal multiplanar reconstruction of the symphysis showing one sublingual midline LF and two submental midline LF (arrows)
- the number and position of lateral foramina, dividing them in four groups on the basis of the relationship with the teeth (incisors, canines, premolars, and molars) (Fig. 3);

**Fig. 3:**
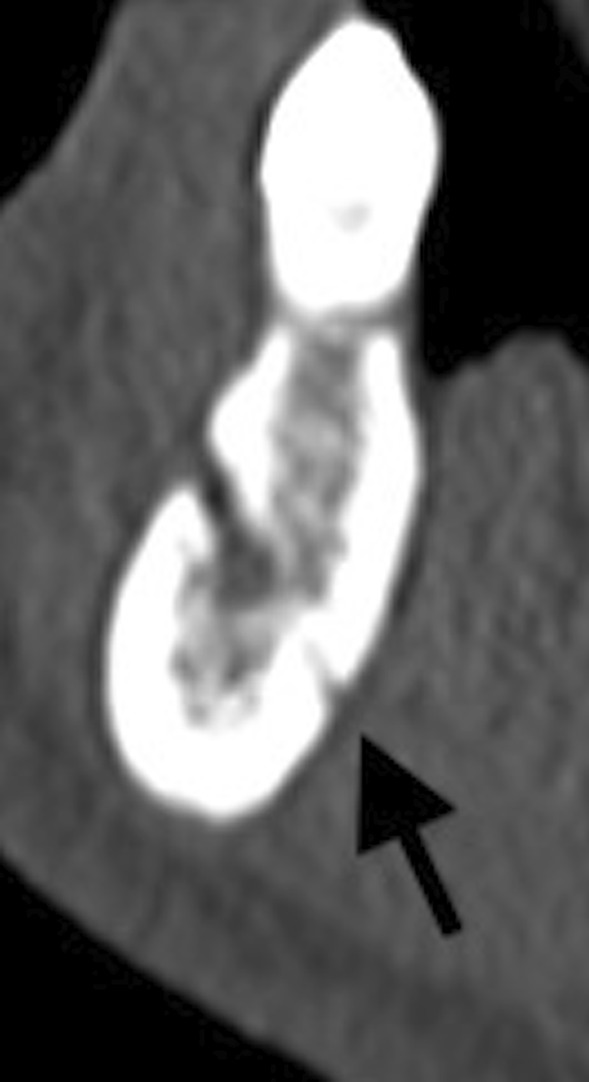
Sagittal multiplanar reconstruction of the left mandibular hemiarch showing one lateral LF in the premolar zone (arrow)
- mandibular shape, given that the mandible cross section can be either normal, or with facial constriction, lingual constriction, and hour glass constriction [13];
- mandibular bone thickness measured in the symphysis and in both hemiarches at the level of the first molars (Fig. 4).

**Fig. 4:**
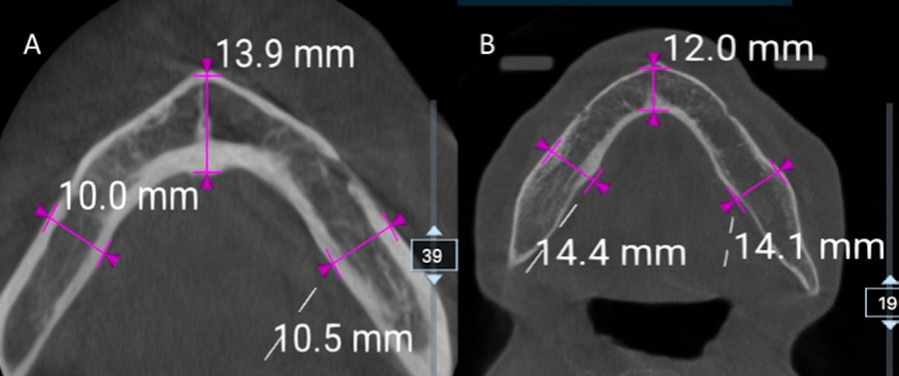
Axial CBCT images of two different patients (**A** and **B**) with mandibular bone thickness measured in the symphysis and in both hemiarches at the level of the first molars

**Fig. 5:**
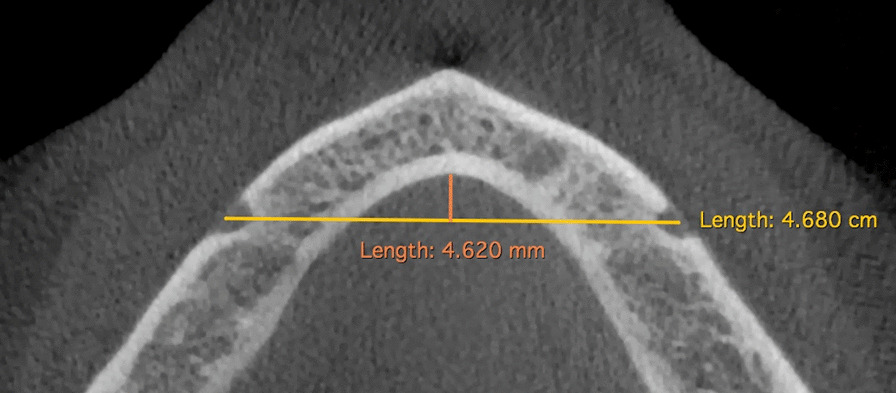
Axial multiplanar reconstruction to estimate the area of the floor of mouth considering it as a triangle. The distance between the mental foramina (base of the triangle) was 4.680 cm and the perpendicular line reaching the symphysis (height of the triangle) was 0.462 cm. The estimated area of the floor of mouth was then calculated obtaining 25.69 cm^2^

To assess the relationship existing between mandibular width and number/position of lateral foramina, we have estimated the area of the floor of mouth considering it as a triangle. To do that, we have calculated the distance between the mental foramina (base of the triangle) on the multiplanar axial reconstruction obtained to see in the same slice both foramina and we have traced a perpendicular line passing reaching the symphysis (height of the triangle). The estimated area of the floor of mouth was then calculated with the following formula as shown in Fig. 5:

$$\frac{{{\text{base}} \times {\text{height}}\;{\text{of}}\;{\text{the}}\;{\text{triangle}}}}{2}$$

The estimated area of the floor of mouth ranged from 17.27 to 30.13 cm^2^. To correlate the evaluation of LF to the width of the mandible, we divided the patients in three groups of 100 on the basis of the estimated area of the floor of mouth: between 17.27 cm^2^ and 22.47 cm^2^ (Group1), between 22.52 and 24.56 cm^2^ (Group2), and between 24.57 and 30.13 cm^2^ (Group3).

### Statistical analysis

We compared the frequency of midline and lateral LF on the basis of gender, mandibular width, shape, and bone thickness using the Chi Square test. For categorical variables, frequencies were provided. Statistical analysis was performed using SPSS® software (v. 26, IBM, Armonk, New York, NY). A *p* value < 0.05 was considered as statistically significant.

## Results

According to our criteria, 300 Caucasian Italian patients (180 males, 120 females; age range 21–87 years) were included in this analysis. The highest frequency per patient observed in our series was of 2 LF (97/300, 32.3%), followed by 3 (81/300, 27%) and 4 (53/300, 17.7%). No LF were observed in 2/300 patients (0.7%), while the highest number of LF (n = 8) was observed in only one patient (0.3%). Regarding midline LF, the highest frequency per patient was of 2 LF (57.3%, 172/300), while the highest number per patient was of 5 LF in just one patient (0.3%) (Fig. 1). The highest frequency of midline LF located above and below the genial tubercle was of 1 in 197/300 patients (65.7%) and in 169/300 patients (56.3%), respectively. Then, concerning lateral LF, the highest frequencies were of 0 (113/300, 37.7%) and of 1 (112/300, 37.3%). We did not observe any statistically significant difference of the number of midline and lateral LF based on gender (*P* = 0.438 and *P* = 0.195, respectively) or mandible width (*P* = 0.069 and *P* = 0.114, respectively). Data concerning the total number and topographic distribution of LF is reported in Table 1, while full data stratified for the mandible width and the relationship of LF with the teeth is reported in Table 2.

**Table 1:** Data concerning the total number and topographic distribution of midline and lateral LF

Total LF				
**0**: 2/300 (0.7%); **1**: 30/300 (10%); **2**: 97/300 (32.3%); **3**: 81/300 (27%); **4**: 53/300 (17.7%); **5**: 29/300 (9.7%); **6**: 6/300 (2%); **7**: 1/300 (0.3%); **8**: 1/300 (0.3%)				
Midline LF				
Total midline LF	Sublingual LF	Submental LF		
**0**: 2/300 (0.7%)	**0**: 2/300 (0.7%)	**0**: 122/300 (40.7%)		
**1**: 68/300 (22.7%)	**1**: 197/300 (65.7%)	**1**: 169/300 (56.3%)		
**2**: 172/300 (57.3%)	**2**: 96/300 (32%)	**2**: 9/300 (3%)		
**3**: 53/300 (17.7%)	**3**: 4/300 (1.3%)			
**4**: 4/300 (1.3%)	**4**: 1/300 (0.3%)			
**5**: 1/300 (0.3%)				

Lateral LF				
Total lateral LF	Incisors	Canines	Premolars	Molars
**0:** 113/300 (37.7%)	**0**: 261/300 (87%)	**0**: 271/300 (90.3%)	**0**: 160/300 (53.3%)	**0**: 284/300 (94.7%)
**1:** 112/300 (37.3%)	**1**: 33/300 (11%)	**1**: 26/300 (8.7%)	**1**: 97/300 (32.3%)	**1**: 14/300 (4.7%)
**2:** 59/300(19.7%)	**2**: 6/300 (2%)	**2**: 3/300 (1%)	**2**: 36/300 (12%)	**2**: 2/300 (0.7%)
**3:** 11/300 (3.7%)			**3**: 7/300 (2.3%)	
**4:** 5/300 (1.7%)

**Table 2:** Data stratified for mandible width and the relationship of LF with the teeth

Total LF	Midline LF	Lateral LF	Incisors	Canines	Premolars	Molars
**Group 1**						
**1:** 11/100	**1:** 31/100	**0:** 38/100	**0**: 89/100	**0**: 91/100	**0**: 61/100	**0**: 93/100
**2:** 40/100	**2:** 52/100	**1:** 47/100	**1**: 8/100	**1**: 8/100	**1**: 32/100	**1**: 5/100
**3:** 29/100	**3:** 17/100	**2:** 14/100	**2**: 3/100	**2**: 1/100	**2**: 6/100	**2**: 1/100
**4:** 15/100		**3:** 1/100			**3**: 1/100	
**5:** 4/100						
**6:** 1/100						
**Group 2**						
**1:** 13/100	**1:** 23/100	**0:** 39/100	**0**: 87/100	**0**: 91/100	**0**: 53/100	**0**: 97/100
**2:** 31/100	**2:** 58/100	**1:** 31/100	**1**: 10/100	**1**: 9/100	**1**: 27/100	**1**: 2/100
**3:** 22/100	**3:** 16/100	**2:** 24/100	**2**: 3/100		**2**: 17/100	**2**: 1/100
**4:** 20/100	**4:** 3/100	**3:** 4/100			**3**: 3/100	
**5:** 10/100		**4:** 2/100				
**6:** 2/100		**5:** 0/100				
**7:** 1/100						
**8:** 1/100						
**Group 3**						
**0:** 2/100	**0:** 2/100	**0:** 36/100	**0**: 85/100	**0**: 89/100	**0**: 47/100	**0**: 93/100
**1:** 6/100	**1:** 14/100	**1:** 34/100	**1**: 15/100	**1**: 9/100	**1**: 38/100	**1**: 7/100
**2:** 26/100	**2:** 62/100	**2:** 21/100		**2**: 2/100	**2**: 12/100	
**3:** 30/100	**3:** 20/100	**3:** 6/100			**3**: 3/100	
**4:** 18/100	**4:** 1/100	**4:** 3/100				
**5:** 15/100	**5:** 1/100					
**6:** 3/100						

The mandible shape was normal in 188 cases, with facial constriction in 42, lingual constriction in 54, and hour glass constriction in 16. The mean bone thickness was 10.76 mm in the symphysis, 10.92 mm in the right hemiarches, and 10.68 in the left hemiarches. No significant differences in the distribution of LF were observed based on mandibular shape and bone thickness (both with *P* > 0.05).

## Discussion

Our main finding is the extreme variability of the number and topographic distribution in midline and lateral LF. The first consideration to be pointed out is that the absence of LF is quite rare but possible, despite several studies have reported at least one LF in their series. It could be postulated that we wrongly missed the LF in those two patients of our series, but also Sekerci et al. [11] and Demiralp et al. [12] found few patients without LF in their series, in 1.8% and 3.4% respectively, thus supporting our results. Concerning the maximum number of LF per patient, we found up to seven LF in one out of 300 patients and eight LF in another patient. Indeed, a large number of LF can be rarely encountered as highlighted by previous studies. Similarly, He et al. found up to seven LF in 2% of their patients [14]. Notably, Patil et al. reported up to 11 LF in 0.5% of their CT study on 300 patients [15]. However, the total number of LF per patient is generally quite lower. In fact, in our series, two LF were observed in 32.3% of patients, three in 27%, and four in 17.7%. These results are in line with those previously published, given that two LF were reported in 28.2% of patients by Sekerci et al. in 34% by Patil et al., and in 32.8% by Demiralp [11, 12, 15]. Conversely, higher variability has been reported concerning the frequency of patients with only one LF that was 10% in our series, similarly to what reported by He et al. (12%) and Demiralp et al. (10.3%) [12, 14], but quite lower than in other series in which one LF has been observed as the most common rate of LF (in up to 40% patients)[15, 16].

Concerning the midline LF, two previous studies confirmed the chance of having no LF in the middle portion of the symphysis with frequencies of 3–3.8% [17, 18]. Nevertheless, no other studies reported more than four LF in the midline, although we found five LF in one patient. We also found four LF in 4/300 patients (1.3%), which is a rare but possible condition also reported by Sheikhi et al. and Wang et al. in 2.9% and 2% of cases, respectively [17, 19]. Further, we reported the highest frequency of patients presenting two midline LF ever published (in 57.3% of cases). Of note, two LF has been the highest frequency of midline LF also in several previous studies, having been observed in 53.9% by Von Arx et al., in 52.9% by Sheikhi et al., in 43.6% by Wang et al., and in 43% by Rosano et al. [5, 17–19]. As a matter of fact, other studies have reported one LF as the highest frequency of LF in the midline, with frequencies reaching 72% [20], while one midline LF was observed in 22.7% of our patients. On the other hand, our frequency of three midline LF (17.7%) is in line with previous studies, in which the frequency of three LF per person has never been reported as the most common rate of midline LF. Regarding the relationship with the genial tubercle, we found that a midline LF above the tubercle is almost invariably detected, similarly to what reported by Tagaya et al. (95%) and Sheikhi et al. (99%) [19, 21]. Further, when a second midline LF is identified, it is located below the genial tubercle in most of the cases. Out of 300 patients, 178 (59.3%) presented at least one midline LF below the genial tubercle, with a slightly lower frequency to that reported by previous studies with frequency ranging from 74.5 to 85% [19, 22].

The frequency of lateral LF reported in literature is quite variable. We observed lateral LF in 62.3% of cases, similarly to Liang et al. (62%) [20], although this frequency was much lower in the series by He et al. (30.1%) and higher in that by Tagaya et al. (80%) [14, 21]. Generally, lateral LF are bilateral and symmetrical, as already reported by previous studies [23]. The total number of LF has been also investigated by Xie et al. who reported one lateral LF in 37.3%, two in 19.7%, three or more in 5,4% [24], in much the same way of our study (one lateral LF in 36.2%, two in 17.7%, three or more in 0,4%). Concerning the relationship between the position of lateral LF and the teeth, we observed 13% of LF in the zone of incisors, 9.8% canines, 46.6% premolars, and 5.4% molars, confirming what already highlighted by other authors on the highest frequency of lateral LF in the zone of premolars [11, 16, 18, 24]. We have resumed in Table 3 the number of LF reported in previous CT and cadaveric studies conducted on different populations.

**Table 3:** Number of LF reported in different CT and cadaveric studies

Study	Country	Sample	LF
Our study	Italy	300 Patients, CBCT	592 Midline, 283 lateral
Von Arx et al. [18]	Switzerland	179 Patients, CBCT	86 Midline, 131 lateral
Trost et al. [25]	Germany	460 Patients, CT	613 Midline, 231 lateral
Sheikhi et al. [19]	Turkey	102 Patients, CBCT	205 Midline
Sekerci et al. [11]	Turkey	500 Patients, CBCT	476 Midline
Liang et al. [20]	Belgium	555 Patients, CT	132 Midline
He et al. [14]	China	200 Patients, CBCT	683
Rosano et al. [5]	Italy	60 Cadaveric mandibles	118
Vandewalle et al. [3]	Belgium	354 Dry mandibles	347

No previous studies have investigated the association of mandible width, shape, and bone thickness with number and distribution of LF, thus a comparison with the literature is not possible. We did not find any statistically significant association with these mandible measurements, as well with gender, although a progressive increase of the number of lateral LF has been observed from patients with smaller mandible width (first group) presenting 78 LF, to those with bigger mandible width (second group with 99 LF and third group with 106 LF), thus some considerations should be pointed out. In the first group, only 1% of subjects showed three or more lateral LF, while in the second and third groups the frequency was 6% and 9%, respectively. Further, considering only the premolars zone, 61% of patients of the first group did not show any lateral LF, as well as the 53% of the second group and the 43% of third.

One of the novelties of this paper is that we attempted to correlate the presence/number of LF to other anatomical aspects, such as the size, shape, and bone thickness of the mandible, which is an innovative point. In addition, the present paper has the strength of presenting data about one cohort of patients belonging to a specific population. In this way, the study could have further knowledge in this specific field. About the clinical relevance, we want to highlight that it is absolutely true that the rate of reported complications in this area is low, but the consequences could be extremely relevant, being among the few causes of death for such interventions. The paper wants to stress the importance to evaluate also such anatomic structures during treatment planning, potentially being as important as other anatomical structures that are commonly considered. Moreover, the evolution of implant techniques, such as the all on four technique, has in recent years drastically increased the number of implants placed in the mandibular symphysis, making this anatomical area one of the most interesting in terms of rehabilitation. Anatomical knowledge of this area also affects other surgical procedures such as biopsies of the oral floor, maxillofacial oncological surgery and emergency surgery for traumas or injuries occurring in the area of the mandibular symphysis. The anatomical variables highlighted in this study lay the foundations for complex diagnoses at the vascular level of the lower jaw in the case of haemorrhages, and are essential for correctly directing emergency triage.

Some limitations of our study should be considered. First, the relatively small sample size of our series, indeed, we cannot exclude that a larger study population would have allowed to obtain a more powerful statistical analysis, even reaching interesting association of the number and anatomic distribution of LF with mandible measurements. Second, we did not evaluate the distance of LF from the alveolar ridge and tooth apex that, in turn, could be an important pre-operative finding to be evaluated before proceeding with implant procedures. Third, the methodology that we used to estimate the mandibular width has not been previously validated, but allowed us to investigate the correlation between the distribution of LF and mandible width. However, it is not felt that this fact was a real limitation, but rather something that deserves further investigation. Last, our CBCT analysis is based on the detection of bone canals, rather than the direct visualization of the vessels. The differences in the number and distribution of LF with some previous studies might be partly related to the different approaches used to assess the LF, for instance through cadaveric skulls which might enable to demonstrate a higher number of LF than CBCT studies. The limited, albeit high, spatial resolution of CT probably might be responsible for a lower detection rate of the LF, although according to several authors CBCT provides highly accurate data concerning mandible anatomy and state that the different frequencies reported in literature is mostly related to the anatomical variability related to different geographical regions. For instance, Rosano et al. [5] found LF in 100% of cases in their cadaveric study, while Tagaya et al. [21] published a double study on five cadavers and 200 patients using CBCT reporting the occurrence of LF in all cadavers and in 95% of patients.

## Conclusions

In conclusion, we have shown the high variability of number and anatomic distribution of midline and lateral LF in a Caucasian Italian group of patients subjected to CBCT without reporting any association with gender and mandible width, shape, and bone thickness. The anatomical variability of the vascular bundles of the floor of mouth must be considered when dealing with surgery in the mandibular region included between the mental foramina to avoid dangerous and life-threating bleeding accidents.

## Data Availability

All data are fully available upon reasonable request. The corresponding author should be contacted if someone wants to request the data.

## Abbreviations

LF: Lingual foramina
CT: Computed tomography
CBCT: Cone-beam CT
